# Distribution of ferritin complex in the adult brain and altered composition in neuroferritinopathy due to a novel variant in the ferritin heavy chain gene *FTH1* (c.409_410del; p.H137Lfs*4)

**DOI:** 10.1111/bpa.13176

**Published:** 2023-06-02

**Authors:** Vincent Umathum, Axel Weber, Daniel Amsel, Ioannis Alexopoulos, Christina Becker, Angela Roth, Andreas Günther, Carmen Selignow, Nadja Ritschel, Anna Nishimura, Alexander Schaiter, Attila Németh, Peter F. M. van der Ven, Till Acker, Anne Schänzer

**Affiliations:** ^1^ Institute of Neuropathology Justus‐Liebig‐University Giessen Giessen Germany; ^2^ Institute of Pathology and Molecular Pathology Bundeswehrkrankenhaus Ulm Ulm Germany; ^3^ Institute of Human Genetics Justus‐Liebig‐University Giessen Giessen Germany; ^4^ Institute for Lung Health Justus‐Liebig‐University Giessen Giessen Germany; ^5^ Center for Infections and Genomics of the Lung (CIGL) Institute for Lung Health (ILH) Justus‐Liebig University Giessen Giessen Germany; ^6^ Institute of Pathology Cytology and Molecular Pathology Wetzlar Germany; ^7^ Department of Pulmonology Agaplesion Evangelisches Krankenhaus Giessen Giessen Germany; ^8^ Centre for Interstitial and Rare Lung Diseases Justus‐Liebig University Giessen Giessen Germany; ^9^ Institute for Cell Biology University of Bonn Bonn Germany

Ferritin is an iron‐binding, spherical protein complex consisting of 24 light (FTL) and heavy (FTH) chain subunits. The proportion of FTL and FTH is determined by tissue and cell differentiation (Figure S1). FTL conveys the iron‐storage function, whereas FTH has a ferroxidase activity centre. In brain tissue, the ferritin complex is primarily composed of FTH.

Hereditary neuroferritinopathy (NF) comprises to a group of neurodegeneration with brain iron accumulation (NBIA, Table S1). NF is an autosomal‐dominant form of NBIA due to a variant in the ferritin light chain gene (*FTL*) on chromosome 19q13.33 [1]. Progressive extrapyramidal movement disturbance, tremor, Parkinsonism and dystonia are typical clinical symptoms with onset in the third to fifth decade of life [2].

A histomorphological hallmark of NF are ferritin containing inclusion bodies (IB), which are predominantly present in the basal ganglia (BG) and the dentate nucleus (DN) of the cerebellum [2, 3].

Here, we present the first variant of ferritin heavy chain gene (*FTH1* c.409_410del) to be associated with NF. With this study, we aim to get a deeper understanding of the function of ferritin in the healthy and diseased brain.

A 78‐year‐old female patient died of a severe COVID‐19 pneumonia. No neurological symptoms were reported at lifetime. There was no family history of central nervous system disease. A 49‐year‐old son was healthy. Post‐mortem brain weight was moderately reduced (1172 g). At coronal sections, BG appeared normal without cystic lesions or atrophy. Histological studies revealed a striking number of IB. Therefore, whole‐exon sequencing at formalin‐fixed paraffin‐embedded brain tissue was performed for analysing genes associated with NBIA.

Genetic analysis revealed a novel heterozygous nonsense variant in *FTH1* [hg19] chr11:61732341_61732342delTG; c.409_410del; p.H137Lfs*4. This variant is not listed in public databases (GnomAD, ExAC, 1000Genomes) and leads to a frameshift and concomitantly to a premature stop‐codon. Artificial Intelligence‐based protein folding algorithm predicts a truncated version of FTH that lacks its carboxy terminal region including a part of helix D and the complete helix E (Figure S2). Importantly, no pathogenic variants in other genes associated with NBIA such as *FTL*, *Pank2*, *Pla2G6*, *C19orf12*, *COASY*, *FA2H*, *WDR45*, *ATP13A2* and *C2orf37* were found.

We were interested to characterise the IB morphology in more detail. IB are round‐shaped eosinophilic structures containing Fe3^+^ deposits, which can be detected by Prussian blue (PB). At resin sections, IB represent distinct ring‐like structures with empty centres. At ultrastructural level, nuclear accumulations of fine‐granular homogeneous material are seen with the chromatin compressed toward the border giving the impression of an outer ring. Cytoplasmic accumulations are also seen (Figure 1A). IB showed a weak expression of ubiquitin. No expression for LC3, p62, AB‐crystallin, and alpha‐synuclein (Figure S3A). IB diameter showed a significant variability in the different brain regions with largest diameter in BG (Figure S4). Interestingly, a high variability of IB was present throughout the brain with the highest number in DN and BG (Figure 1B and Table S2). In the cortex, IB were obtained only in the grey matter, with no inclusions present in the neurons (Figure S3B).

**FIGURE 1:**
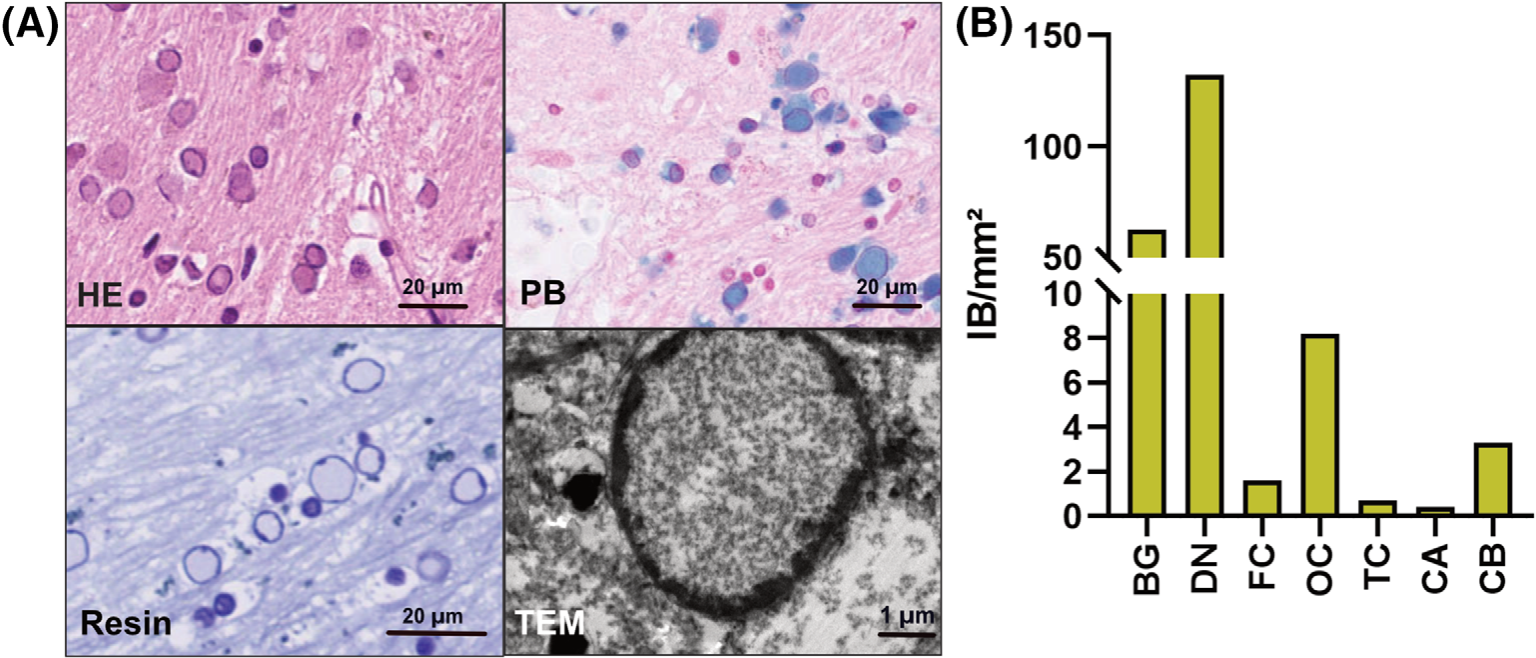
Morphological characterisation of inclusion bodies. In H&E‐stained sections, numerous IB are present in the basal ganglia. IB show a strong blue staining with PB indicating Fe^3+^ deposits. In methylene‐blue stained resin sections, IB have a sharp ring‐like border with a pale homogeneous centre. At electron microscopy, IB show fine granular material with lateralised, condensed chromatin, indicating that IB correspond to enlarged nuclei. In the cytoplasm, similar granular material is seen (A). A high variability of IB distribution is present with highest number in DN, BG and OC (B). BG, basal ganglia; CA, hippocampal formation; CB, cerebellar cortex; DN, dentate nucleus; FC, frontal cortex; IB, inclusion bodies; OC, occipital cortex; PB, Prussian Blue; TC, temporal cortex.

In addition, we performed a detailed confocal laser‐scanning microscopy analysis with immunofluorescence antibodies against FTL, glial fibrillary acid protein (GFAP) and nuclear stain 4′,6‐diamidino‐2‐phenylindole (DAPI). In control, FTL was mainly expressed in the cytoplasm with only weak nuclear expression (Figure 2A). In NF‐brain, nuclei were enlarged with strong FTL expression, while nuclear chromatin (DAPI) was present as an outer ring structure. These findings indicate that IB correspond to enlarged nuclei due to ferritin complex accumulation. Most cells containing IB did not stain for the glial marker GFAP, suggesting an oligodendroglial cell phenotype (Figure 2B). 3D rendering underlines these findings (Video S1). However, neither cytoplasmatic nor extracellular IB were observed in our study (Figure S5).

**FIGURE 2:**
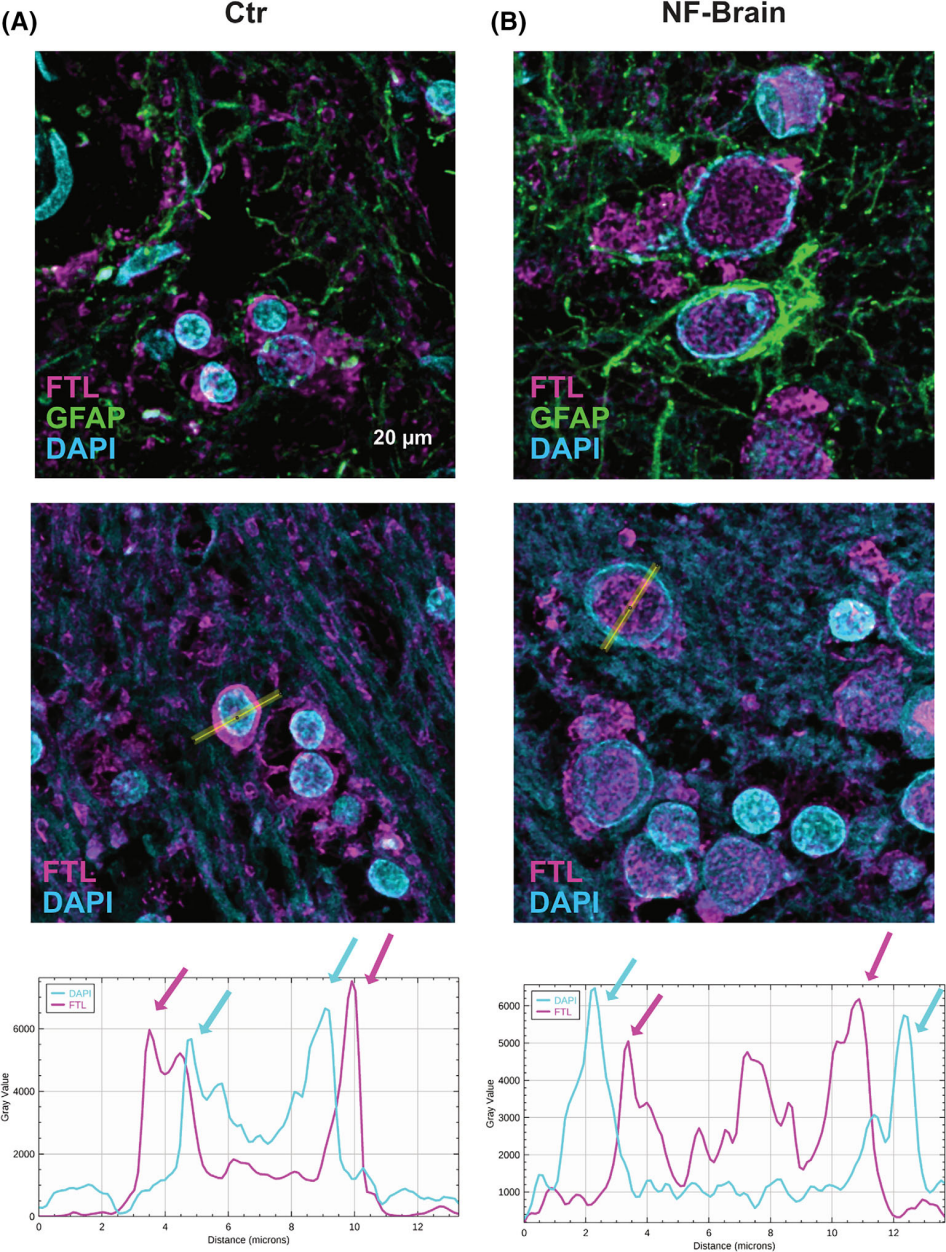
Inclusion bodies show strong accumulation of ferritin complex expression of FTL. Analyses of FTL expression in control (Ctr) compared to NF‐brain in the BG using confocal laser microscopy shows that in Ctr FTL is mainly expressed in cell cytoplasm. Linear intensity plot confirms strong perinuclear FTL (pink arrows) with lower nuclear FTL expression. Nuclear stain DAPI is indicated by blue arrows (A). In NF‐brain, the IB show strong nuclear staining of FTL with enlargement of the nuclear diameter. Most of these cells are GFAP‐negative indicative for oligodendrocytes. With linear intensity plot nuclear FTL expression levels are high with expansion of the nuclear diameter (pink arrows). DAPI staining presents as an outer ring resembling remnant of chromatin (blue arrows) (B). The findings are demonstrated by FTL 3D reconstruction (Video S1). DAPI, 4′,6‐diamidino‐2‐phenylindole; FTL, ferritin light chain; IB, inclusion bodies; NF, neuroferritinopathy.

Few extracerebral IB were found in the kidney (tubulus epithelium) and in the liver (hepatocyte). These findings are consistent with literature from patients with NF due to *FTL* variants, suggesting NF as a systemic disease similar to Parkinson's disease [4, 5] (Figure S6).

Moreover, we were interested to estimate the distribution of the ferritin complex in the diseased brain compared to three gender and age matched controls with morphometric methods. In NF‐brain, extracellular and intracellular FTH expression was significantly higher compared to controls (Figure 3A,B). Distribution of ferritin in different brain areas revealed highest FTH expression in the DN of the cerebellum in controls. In NF‐brain, FTH expression was highest in DN and BG. In cortical areas (frontal, occipital, temporal, cerebellar) and hippocampus, FTH expression was lower in controls compared to the NF‐brain (Figure 3C and Table S3).

**FIGURE 3:**
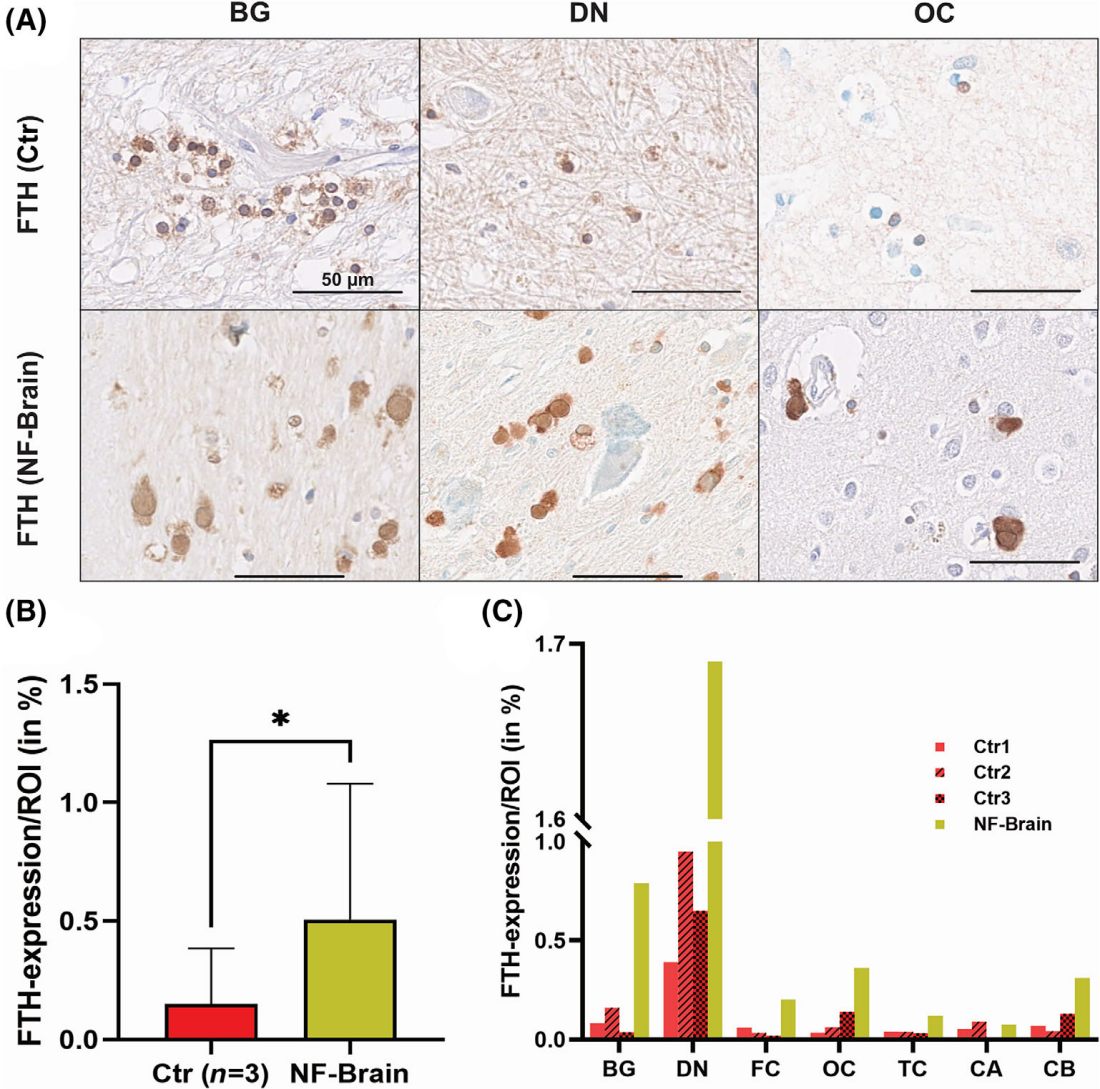
FTH expression is increased in NF‐brain with variable distribution in different brain areas. Analyses of FTH expression in controls (Ctr) compared to NF‐brain in different brain areas show that in Ctr FTH deposits are mainly seen in BG and DN, with lower expression in cortical areas OC. In NF‐brain, strong FTH deposits are observed in all brain regions (A). Morphometric analysis revealed a significant higher FTH expression in NF‐brain (0.51%) compared to Ctr (0.15%, *p* = 0.03) (B). Comparing the different brain regions strongest differences of FTH expression are seen in DN, BG and OC (C). BG, basal ganglia; CA, hippocampal formation; CB, cerebellar cortex; DN, dentate nucleus; FC, frontal cortex; FTH, ferritin heavy chain; NF, neuroferritinopathy; OC, occipital cortex; TC, temporal cortex.

Analysing the cellular distribution of ferritin complex showed that in controls FTH is mainly located in the nucleus whereas FTL is more likely to be found in the cytoplasm. In NF‐brain, a strong nuclear but also cytoplasmatic co‐expression of both FTH and FTL was observed (Figure 4A). As indicated by the intensity level (ratio FTH/FTL), a significant increase of nuclear and cytoplasmatic FTH in NF‐brain compared to controls were seen (Figure 4B,C). However, the expression of both FTH and FTL was higher in NF brain compared to controls (Figure 4D).

**FIGURE 4:**
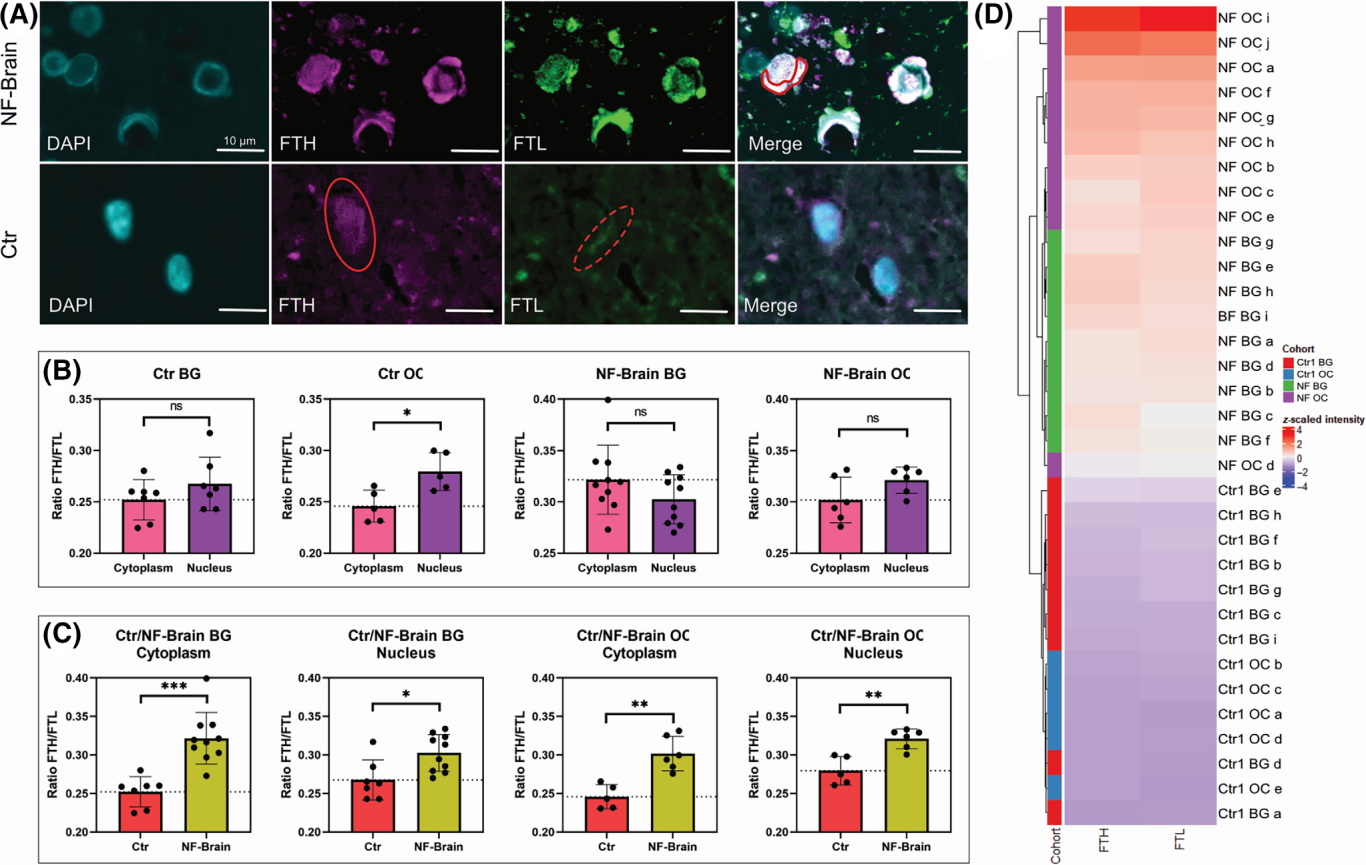
Cellular distribution of FTH and FTL is altered in NF‐brain. Analyses of co expression of FTL and FTH expression in control (Ctr) compared to NF‐brain in the basal ganglia at cellular level shows that in NF‐brain co‐expression of FTH and FTL is mainly seen nuclear (IB) but also in the cytoplasm (red line). In Ctr FTH is mainly expressed in the nucleus (red ellipse) whereas FTL is more highly expressed in the cytoplasm (red dotted ellipse) (A). In Ctr, the proportion of FTH is higher in the nucleus than in the cytoplasm. In contrast, in the NF‐brain no significant differences are seen between nuclear and cytoplasmic FTH expression (B). Estimating the intensity level (ratio FTH/FTL), in NF‐brain FTH is significantly increased in the nucleus and cytoplasm compared to Ctr (ns = *p* > 0.05; *p < 0.05; **p < 0.01; ***p < 0.001) (C). Unsupervised clustering of NF‐brain (OC, BG) and Ctr (OC, BG) clearly show that the intensity of FTL‐ and FTH‐expression in single cells is higher in NF‐brain compared to Ctr (D). BG, basal ganglia; FTH, ferritin heavy chain; FTL, ferritin heavy chain; IB, inclusion bodies; NF, neuroferritinopathy; OC, occipital cortex.

For detailed “Material and Methods,” see Supplemental Data 4.

In summary, we report the first NF due to a novel variant in the ferritin heavy chain gene (*FTH1*). Based on the distinct neuropathology with ferritin‐positive IB, the absence of the variant in the healthy population and the absence of likely pathogenic variants in other genes associated with NBIA, we propose that the variant in *FTH1* (c.409_410del; p.H137Lfs*4) is causative for the pathological findings in our patient. Importantly, the neuropathological findings are highly compatible with NF caused by *FTL* variants [2, 3].

The variant of *FTH1* found in our patient (c.409_410del; p.H137Lfs*4) is predicted to lead to expression of a protein in which the carboxyterminal 47 amino acids are replaced by the amino acids leucine, proline and glutamic acid (LPE). Initiation of nonsense‐mediated mRNA decay is highly improbable since the premature stop‐codon caused by the *FTH1* c.409‐410del variant is located in the last exon. Therefore, the expression of a truncated protein that lacks parts of the protein important for normal assembly of the ferritin complex is highly probable, whereas effects on its stability cannot be inferred [6].

In our study, we further characterised the morphology and distribution of IB in the diseased brain in detail. We demonstrate that IB consist of intranuclear ferritin accumulations leading to enlarged nuclei. The distribution of IB in our patient are consistent with the distribution in NF due to the *FTL* with highest number in basal ganglia and dentate nucleus of cerebellum. Interestingly, the number of IBs seems to correlate with the physiological distribution of iron in the ageing brain, where iron is preferentially found in the BG. This might explain the enhanced distribution of IB especially in BG [7]. A detailed analysis of the ferritin complex subunits at cellular level has not been described in human brains so far. In mice, it has been demonstrated that FTH is highly expressed in the nucleus, whereas in the cytoplasm FTL expression is stronger [8]. With immunofluorescence studies, we can confirm these observations in one control brain. However, in our diseased brain (*FTH1*), there was a significant increase in FTH and FTL in both cytoplasm and nucleus, with FTH accumulating disproportionately. In addition, IB consisted of enlarged nuclei due to accumulation of ferritin complex of FTH and FTL. These findings may indicate that accumulation of FTH in NF brain (*FTH1*) is due to pathological ferritin complex formation and impaired iron metabolism with impaired protein function in line with the role of pathological protein accumulation as seen in other neurodegenerative diseases [4, 7]. The primary nuclear pathology of the *FTH1* variant would be in line with the predominantly nuclear localization of FTH as seen in the control brains. Consistent with this notion, in NF with *FTL* variants, the dysfunctional ferritin complex is leading to cellular accumulation of iron and ferritin accompanied increased FTL levels [3]. However, in these patients detailed analysis of FTL/FTH ration in the ferritin complex has not been performed.

Analysing the function of ferritin complex in animal models shows, that in *Fth* ^−/−^ knockout mice FTH deficiency leads to early embryonic lethality. This indicates that the loss of FTH cannot be compensated by the FTL subunit and results in dysfunctional ferritin complex [9]. In comparison, in *Ftl* ^−/−^ knockout mice, the storage function of ferritin can be partly rescued by FTH, resulting in partial embryonal survival [10]. Taken together, these findings may indicate that *FTH1* variant has residual activity for iron storage and that the ferritin complex is dysfunctional but not completely without function.

In conclusion, we describe the first patient with NF due to a variant in *FTH1* (c.409_410del; p.H137Lfs*4). Morphological characterisation of IB, a histomorphological hallmark of NF, revealed that IB consists of enlarged nuclei containing ferritin accumulation. In addition, the distribution and composition of ferritin complex is altered in the NF brain compared to controls. These findings may contribute to a better understanding of ferritin complex function in normal and diseased brain.

## AUTHOR CONTRIBUTIONS

VU, TA and AS designed the study, analysed the data and wrote the manuscript. AR performed immunofluorescence studies. DA, CS and AW performed genetic analysis. IA performed CLSM analysis. ANS performed morphometric analysis. ASch performed data analysis. Tissue collection and performed autopsy was done by CB. Clinical data was provided by AG. PVV discussed genetic data and corrected the manuscript. AN and NR performed molecular analysis. All authors have read and approved the final manuscript.

## CONFLICT OF INTEREST STATEMENT

The authors declare no conflicts of interest.

## ETHICS STATEMENT

Written informed consent was obtained from the patient's relatives. This project was approved by the local ethics committee (AZ69/20, Justus‐Liebig‐University Gießen) and conducted in accordance with the Declaration of Helsinki.

## Supporting information


**Data S1.** Supporting Information.

**Video S1.** 3D rendering of confocal laser‐scanning microscopy (CMSL) analysis.

## Data Availability

Availability of data and material. The datasets supporting the conclusions of this article are included within the article and its supporting information.
