# Supplementary material for: Distribution of ferritin complex in the adult brain and altered composition in neuroferritinopathy due to a novel variant in the ferritin heavy chain gene FTH1 (c.409_410del; p.H137Lfs*4)
Source: Brain Pathol. 2023 Jun 2;34(1):e13176. doi: 10.1111/bpa.13176 (PMC10711253; doi:10.1111/bpa.13176)
Supplement: Supplementary file 1 — Data S1. Supporting Information. [file BPA-34-e13176-s001.pdf]

Supplemental Data 1:

Figure S1

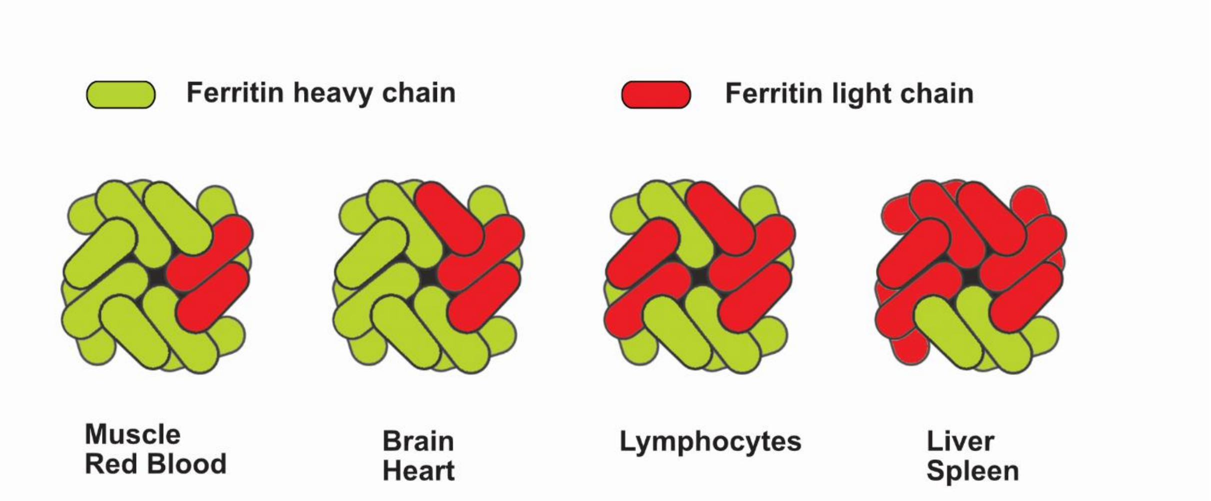

Figure S1: Ferritin Complex composition

The composition of the ferritin complex depends on cell type and organ. In organs with a high iron demand, e.g. muscles and brain, the proportion of FTH with its ferroxidase activity is higher. In the liver and spleen, ferritin consists mainly of FTL due to its storage function. Modified from Harrison and Arosio [6].

Figure S2

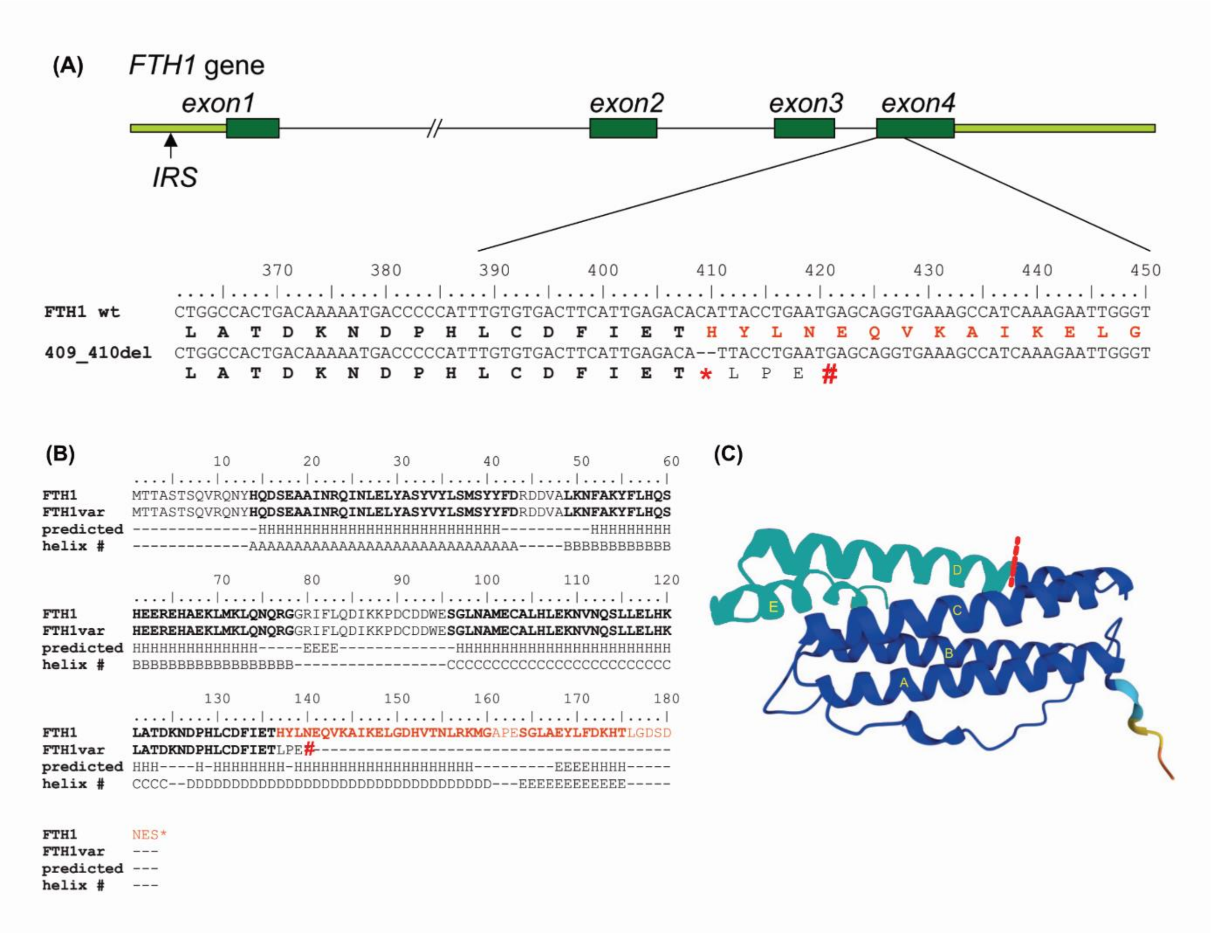

Figure S2: Novel nonsense variant in *FTHl* is causative for NF

WES of FFPE brain sample revealed on exon 4 a deletion of cytosine and adenine (red *) at position 409 and 410. The shift leads to a stop codon UGA (red #) (A). The truncated protein completely lacks helix E and parts of helix D (B). 3D protein prediction of the ferritin heavy chain shows the truncated protein in dark blue; the termination of FTH synthesis is indicated by the red line and missing helices are shown in turquoise (C). WES = Whole exon sequencing, FFPE = formalin fixed paraffin embedded

Figure S3

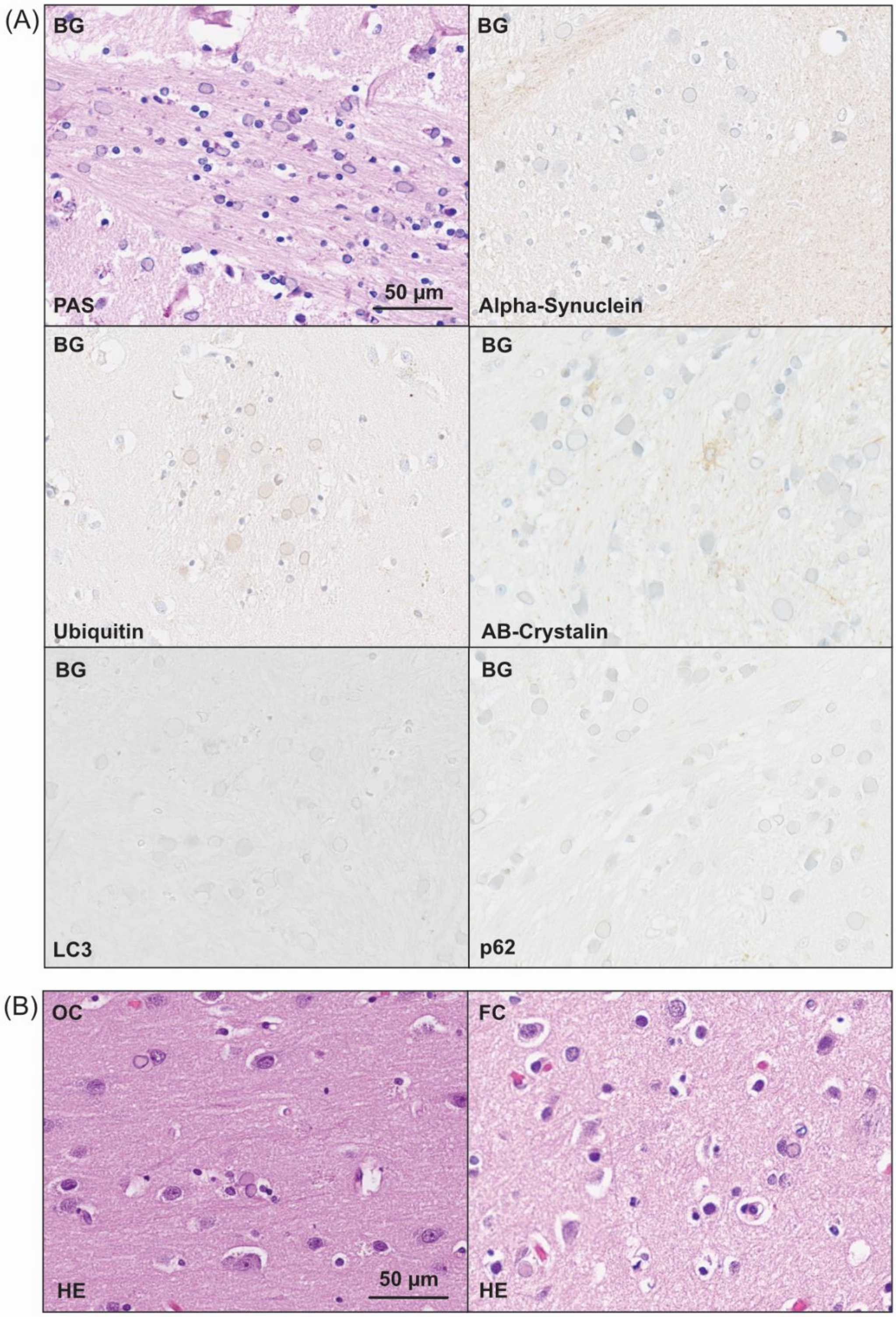

(A)
BG
PAS
50 µm
BG
Alpha-Synuclein
BG
Ubiquitin
BG
AB-Crystalin
BG
LC3
BG
p62
(B)
OC
HE
50 µm
FC
HE

Figure S3: Histopathology of NF-brain and inclusion body characterization

IB are negative for PAS, alpha-synuclein, AB-crystallin, LC3 and p62. Ubiquitin is weakly expressed (A). In the cortical areas, IB are only visible in the grey matter (B).

Figure S4

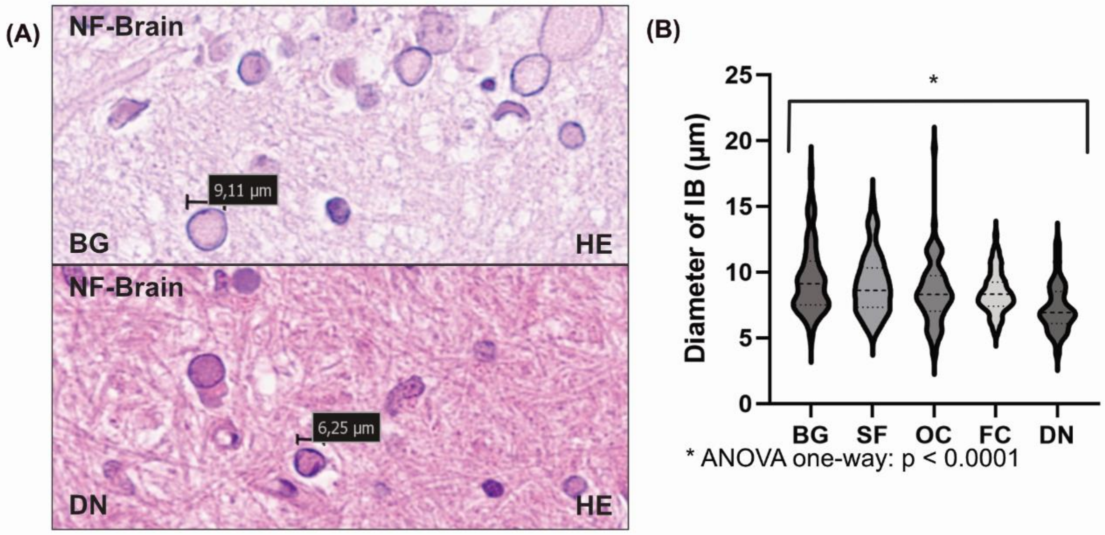

(C) Size of IB in different brain regions

(µm)	BG	SF	OC	FC	DN
Minimum	5.2	5.5	3.8	5.5	3.9
25% Percentile	7.5	7.3	7.0	7.4	6.1
Median	9.1	8.6	8.3	8.3	6.9
75% Percentile	10.8	10.3	9.8	9.2	8.5
Maximum	17.5	15.3	19.4	12.8	12.4

Figure S4: Variability of inclusion body diameter in different brain regions

HE stained sections with IB in BG and DN (A). The comparison of the median IB diameters in different brain regions is statistically significant (ANOVA one-way test; $p < 0.0001$). An unpaired t-test was performed to compare the data from BG and DN. The analysis shows statistical significance between these two groups ($p = < 0.0001$) (B). Descriptive statistics of IB diameters (C).

Figure S5

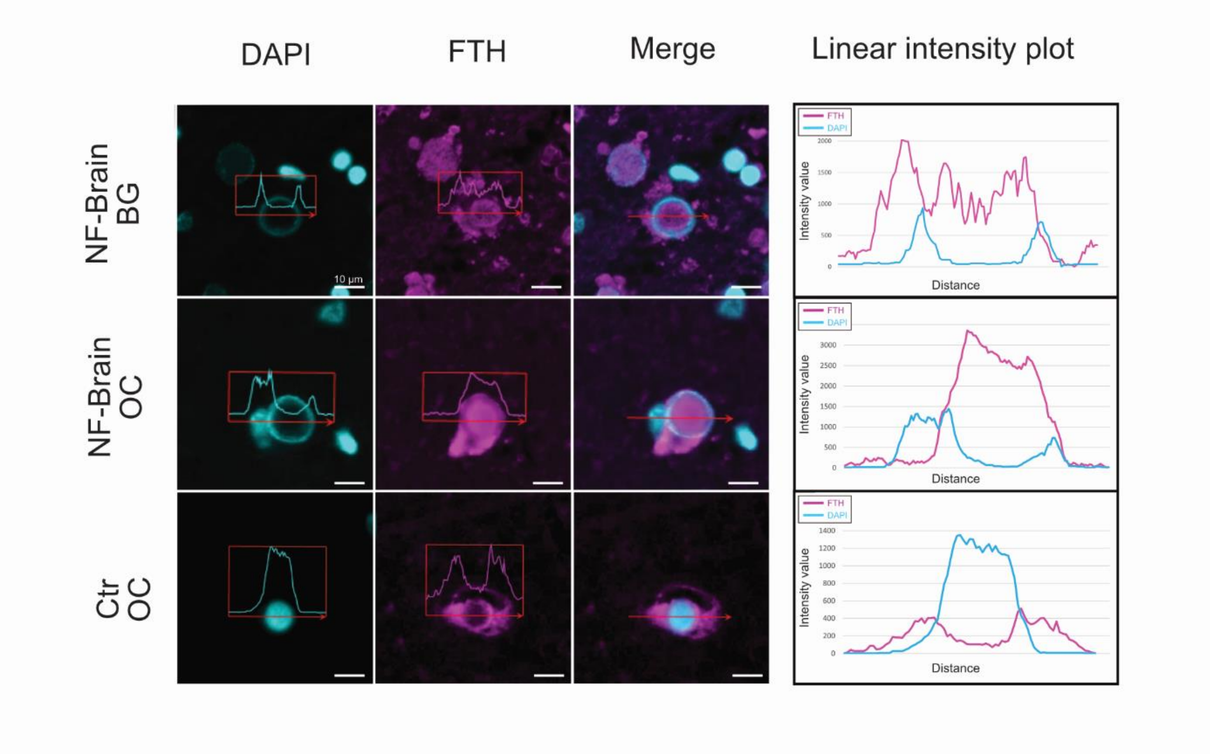

Figure S5: Expression of FTH and FTL in NF-brain compared to control.

In NF-brain nuclear expression of FTH is present with ring like DAPI staining corresponded to IB (upper row). IB which are located directly perinuclear, suggesting cytoplasmic localisation, also show a background signal for DAPI, consistent with IB. A nucleus without an accumulation of FTH is located in the direct proximity of the IB on the left (middle row). In Ctr, perinuclear expression of FTH is visible (lower row). Linear intensity plots clearly show nuclear accumulation of FTH in IB, while DAPI only shows a background signal with increases towards the nuclear membrane. Enhanced cytoplasmic FTH expression is also seen to the left of the nucleus (upper row). The DAPI background signal of the IB in the middle row demonstrates that this IB is indeed an expanded nucleus and not a cytoplasmic inclusion. In Ctr, intranuclear FTH intensity values are low and DAPI signals are high. Cytoplasmic FTH accumulations can be seen (lower row).

Figure S6

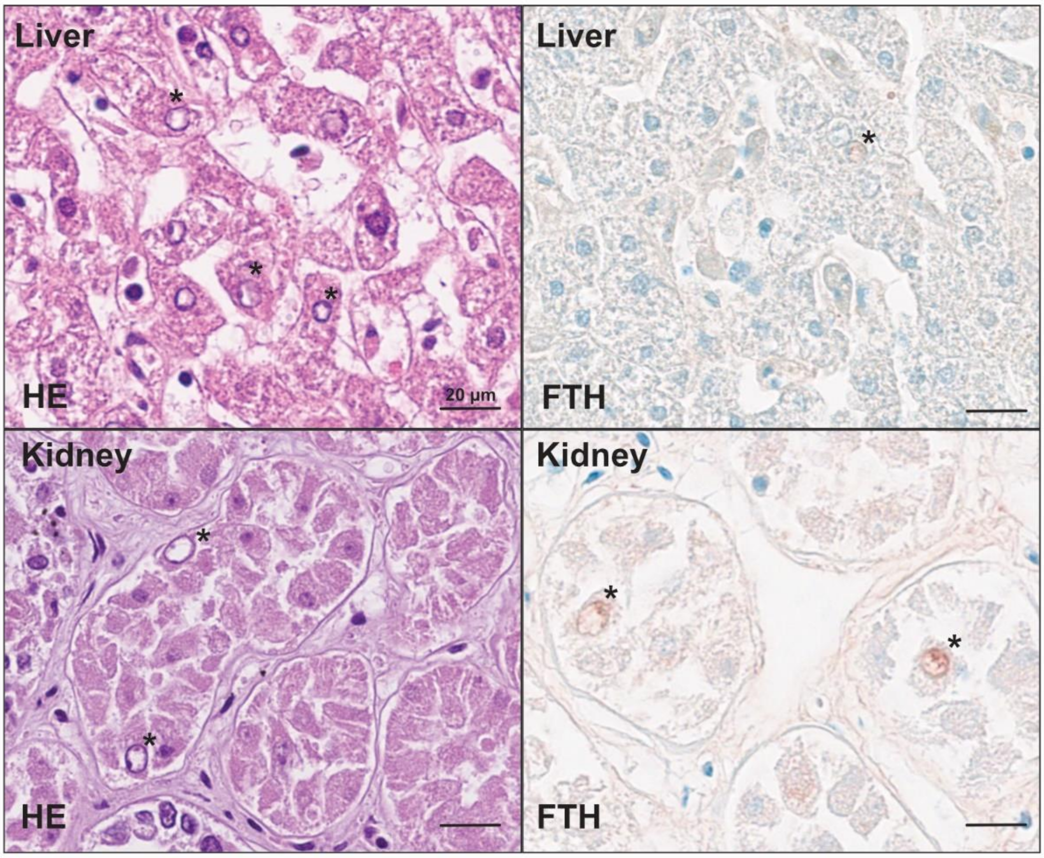

Figure S6: Extracerebral inclusion bodies

HE stained liver sections reveals nuclear IB (asterisks) in hepatocytes. Due to autolysis the immunohistochemical staining of IB for FTH is weak (asterisks). In HE stained kidney sections IB can be clearly seen (asterisks) although the tubular epithelia are autolytic. Immunohistochemical staining for FTH identifies nuclear IB in the tubular epithelium of the kidney (asterix).

Supplemental Data 2:

Movie S1:3D rendering of confocal laser-scanning microscopy (CMSL) analysis

Supplemental Data 3:

Table S1: Overview of subtypes of neurodegeneration with brain iron accumulation (NBIA)

NBIA nomenclature	Disorder	Gen	Neuropathological findings	Clinical presentation	Inheritance
NBIA 1	Pantothenate kinase-associated neurodegeneration (PKAN)	*PANK2*	"mummified" neurons, axonal degeneration, and iron accumulation in globus pallidus	mean age of onset: 3 years; Dystonia; mild global developmental delay and dyspraxia; Pigmentary retinopathy	AR
NBIA 2a	Phospholipase A_2-associated neurodegeneration (PLAN), including infantile neuroaxonal dystrophy (INAD)	*PLA2G6*	Lewy-Bodies and neurofibrillary tangles	Mean age of onset: 6 months to 3 years; developmental regression, hypotonia, and spastic tetraparesis; Visual impairment is common	AR
NBIA 2b	PLA2G6-related neuroaxonal dystrophy, atypical	*PLA2G6*		onset in adolescence or early adulthood; dystonia-parkinsonism; mild intellectual impairment	AR
NBIA 3	Hereditary neuroferritinopathy	*FTL*	Inclusion Bodies of ferritin	Mean age of onset around 40 years; Chorea and dystonia are the most common initial presentations, followed by tremor, parkinsonism, cerebellar ataxia, psychiatric symptoms and tics.	AD
NBIA 4	Mitochondrial membrane protein-associated neurodegeneration (MPAN)	*C19orf12*	Neuronal loss, widespread iron deposits, and eosinophilic spheroidal structures in the basal ganglia. Lewy bodies.	begin in childhood or early adulthood; movement abnormalities, behavioral disturbances, and dementia; Dystonia, spasticity, and parkinsonism are common in early disease	AD, AR
NBIA 5	Beta-propeller protein-associated neurodegeneration (BPAN)	*WDR45*	Axonal spheroids neurofibrillary tangles	Onset in infancy and childhood; Language skills are limited, expressive language may never be acquired. Midline hand stereotypies are common;	XLD

				later: movement disorder, dystonia or parkinsonism	
NBIA 6	CoA synthase protein-associated neurodegeneration (CoPAN)	*COASY*	Iron accumulation and calcification in basal ganglia	Onset in childhood; dystonia, spasticity with cognitive impairment, parkinsonism, obsessive-compulsive disorder and peripheral neuropathy.	AR
NBIA 7	Neurodegeneration with brain iron accumulation 7	*REPS1*			AR
NBIA 8	Neurodegeneration with brain iron accumulation 8	*CRAT*			AR

Note: AR = autosomal-recessive, AD = autosomal-dominant, XLD = X-linked dominant. Modified from Gregory and Hayflick [3].

Table S2: Number of IB in different brain regions

Region	BG	DN	FC	OC	TC	CA	CB
IB/mm²	62.6	132.2	1.6	8.2	0.7	0.4	3.3

Table S3: FTH expression in NF-Brain and Ctr

FTH-expression	FC	OC	TC	CA	DN	CB	BG
Ctr, n=3 (in %)	0.038	0.079	0.037	0.052	0.663	0.08	0.093
NF-Brain (in %)	0.2	0.36	0.12	0.07	1.69	0.31	0.79
Ratio (Ctr:NF-Brain)	1 : 5.3	1 : 4.6	1 : 3.2	1 : 1.4	1 : 2.5	1 : 0.8	1 : 8.5

Table S4: Patients characteristics

	Age (years)	sex	Post-mortem interval (days)	Cause of death	Co-morbidities	Neuropathological finding
**NF-Brain**	76	f	3	Covid-19 associated pneumonia	none	Mild gliosis and oedema, inclusion bodies
**Ctr 1**	88	m	13	MOF (pulmonary adeno-carcinoma)	Arterial hypertension, oro-pharyngeal SCC	Mild gliosis and oedema.
**Ctr 2**	88	f	9	MOF	Chronical renal failure, heart failure, arterial hypertension	Mild gliosis and oedema.
**Ctr 3**	69	f	4	ARDS	Idiopathic pulmonary fibrosis, usual interstitial pneumonia	Mild gliosis and oedema.

Note: MOF = multi organ failure, ARDS = acute respiratory distress syndrome, SCC = squamous cell cancer

Supplemental Data 4

Material and methods

Clinical data

A 78-year old female patient died due to a COVID19 infection. An autopsy was performed and tissue samples were taken and fixed in 4% buffered formalin fixed and embedded in paraffin (FFPE) for further investigations. The brain (NF-brain) was submitted to further neuropathological investigations. In addition, tissue from three age matched control brains (Ctr) from subjects without clinical and histomorphological evidence of neurodegenerative disease were included in the study. See detailed patients' characteristics in table S4, supporting information.

Genetic analysis

Whole exome sequencing (WES) from FFPE CNS tissue of the patient was performed using short-read-sequencing technology to detect genetic variations in the patient's DNA. Hybridization capture-based target enrichment (AGILENT SureSelect Enzymatic Fragmentation Kit und SureSelect XT HS Target Enrichment revA, SureSelect Human All Exon v7) was done fully automated (AGILENT Magnis NGS Prep System). Library QC by dsDNA HS Assay on Qubit®3.0 Fluorometer (Thermofisher) and Agilent DNF-915 Reagent Kit (35-5000bp) on Fragment Analyzer 12 Capillary System 33cm (Agilent). Analysis was performed on Illumina NovaSeq Platform. The coverage greater 10x over the entire exomes was 93.72% and the mean target coverage was 53.33x. The maximum target coverage was 1,855x. The evaluation is carried out by means of an in-house germline pipeline based on BWA [9] aligned to the National Center for Biotechnology Information human reference genome (GRCh37/hg19), GATK 4.2.0 [10] and Gemini [11]. Protein structure prediction was made by alpha fold [8, 12]

RNA analysis

RNA was isolated from FFPE tissue using the RNeasy FFPE Kit (QIAGEN). Quality of the RNA solution was assessed using the High Sensitivity RNA ScreenTape on a TapeStation device (Agilent) by calculatingand the RNA Integrity Number (RIN) was determined. RNA was reversely transcribed into cDNA using Maxima H Minus First Strand cDNA Synthesis Kit (Thermo Fisher Scientific). Real-time PCR was performed using TaqMan Gene Expression

Array Assays to detect FTH1 (assay ID Hs01000476-g1 and Hs01000478_g1), FTL (assay ID Hs00902546_g1), GAPDH (assay ID Hs00266705_g1) and TUBB (assay ID Hs03929064_g1) with amplicon sizes of maximum 86 base pairs on a QuantStudio 3 Real-Time PCR System (Thermo Fisher Scientific). Control brain tissue-derived RNA was used for comparison.

Histopathology

Tissue samples for histopathology were taken from all organs and representative brain areas and processed using standard procedures. From FFPE samples 4 µm thick sections were stained for hematoxylin and eosin (HE) (MEDITE Medical GmbH, Burgdorf, Germany), Prussian Blue (PB), periodic acid–Schiff reaction (PAS) (both at Ventana Bench Mark Special Stains; Roche, Mannheim, Germany). Additional FFPE sections from extra cerebral organs (liver, kidney, lungs and heart) were stained for HE and PB.

Immunohistochemical studies

Immunohistochemistry was performed at 4 µm thick FFPE sections of selected brain regions from the patient and three control patients using an automated BenchMark XT staining platform (Ventana, Heidelberg, Germany, ultraview universal DAB detection kit). Primary antibodies against glial fibrillary acidic protein (GFAP, rb-polyclonal, Abcam, ab16997, 1:100), ferritin-light-chain (FTL, ms-monoclonal, Abcam, ab218400, 1:1000), ferritin-heavy-chain (FTH, rb-polyclonal, Invitrogen, PA5-27500, 1:8000), Beta-Amyloid (ms-monoclonal, Biolegend, 800710, 1:20.000), AT8 (ms-monoclonal, Invitrogen, MN1020, 1:200), LC3 (ms-monoclonal, Biotin, 0231-100, 1:100), Ubiquitin (rb-polyclonal, Zytomed, 521-3354, 1:50), AB-Crystallin (ms-monoclonal, Abcam, 13496, 1:5000), Alpha-Synuclein (ms-monoclonal, Invitrogen, 180215, 1:50) and p62 (ms-monoclonal, BD Bioscience, 610832, 1:500) were used. From extracerebral organs, (liver, kidney, lungs and heart) sections were stained with antibodies against FTL and FTH. Sections were analysed at a Leica microscope (Leica DM LB, Wetzlar, Germany).

Immunofluorescence studies

Immunofluorescence staining was performed on 4 µm thick FFPE sections of selected brain regions from NF-Brain and one Ctr brain with antibodies against glial fibrillary acidic protein (GFAP, rb-polyclonal, Abcam, ab16997, 1:100), ferritin-light-chain (FTL, ms-monoclonal, Santa Cruz, sc-74513, 1:50), ferritin-heavy-chain (FTH, rb-polyclonal, Invitrogen, PA5-27500, 1:100). Applied secondary antibodies were gt-Alexa Fluor 568 anti-mouse (Invitrogen, red), gt-

Alexa Flour 568 anti-rabbit (Invitrogen, red) and gt-Alexa Fluor 488 anti-mouse (Invitrogen, green). Sections were mounted with Fluoroshield mounting medium with DAPI (Abcam).

Morphometric analysis

IB morphology and FTH deposits were analysed on whole slide images (WSI) using NDPview2 at digitalized sections (Hamamatsu NanoZoom) [13]. Items per region of interest (ROI) were counted using QuPath software [1]. The distribution of the IB was analysed at PB stained sections in representative NF-brain areas: frontal cortex (FC), occipital cortex (OC), temporal cortex (TC), hippocampal formation (CA), dentate nucleus (DN) cerebellar cortex (CB), basal ganglia (BG). Number of IB were expressed in IB/mm². The variation of diameter of the IB was measured at HE stained sections in different NF-brain regions: striato-pallidal fibres or pencil bundles of Wilson (SF), BG, FC, OC and DN. Variation of IB size was expressed in mm. FTH positive deposits were analysed at FTH-DAB stained sections in NF-brain and three Ctr-brains in BG, DN, FC, OC, TC, CA and CB. The ROI was chosen automatically by the tissue detection of the QuPath software. Artefacts as sections folds and scratches were excluded. Positive pixel count was done by using threshold detection for the FTH-expression (DAB). Positive pixel/ROI were expressed in %. The ratio of FTH Ctr / FTH NF-Brain was estimated to compare the FTH expression in controls and in NF-Brain.

FTH and FTL expression level were analysed on immunofluorescence-stained sections of the NF brain and Ctr brain 1 at BG and OC. Sections were digitalized using Zeiss Axio Scan Z1. The scanner was equipped with a colibri.2 fluorescence light source and images were scanned with Plan Apochromat objective (20x). To assess the intensity of nuclear and cytoplasmatic FTH and FTL expression, the intensity values were determined with a linear intensity plot and evaluated per cell using ZEN 3.6 blue edition software [14]. The mean value of the individual measurement points was determined for each cell (in average 9 cells per area). The ratio of nuclear and cytoplasmatic expression of FTH/FTL were calculated. Figures were created with GraphPad Prism (version 9.4.1).

Confocal laser-scanning microscope (CLSM)

Additional analysis was performed at images of immunofluorescence-stained sections with antibodies against FTL and GFAP, captured by an inverted confocal laser-scanning microscope (Leica SP8, Leica Microsystems, Germany), controlled with the LASX software (version 5.5.7), using a 20x NA 0.75 Plan-Apochromat Air objective. The z step size of all acquisitions was kept the same at 0.545 um. DAPI was excited with a 405nm laser (set at 0.6%). For the

excitation of the AF488 (GFAP) and AF568 (FTL) a 499nm at 7.3% and a 577nm at 1.3% laser line was used, respectively, using a White Light Laser (WLL). The nominal laser power of the WLL was previously set at 50%. All three fluorescence emissions were captured using three Hybrid Detectors (set at 100%-Standard mode). The acquisition was completed in a line-by-line sequential manner, using 2x line averaging at 600 lines per second. The pinhole size was set at 0.8AU (calculated for 539nm of wavelength). The acquired data were deconvolved with the Leica Lightning software module, using an adaptive approach. The final figures were generated using the OMERO.figure module. The 3D renderings were generated using the 3D module of the LAS X software, while for the line profiles we used the ImageJ/ Fiji software [7] .

Transmission Electron Microscopy (TEM) and resin sections

Small samples from the BG area of the formalin fixed NF brain were processed for electron microscopy according to standard procedures [2]. Semithin resin sections were stained with Richardson and analysed at a Leica microscope (Leica DM LB, Wetzlar, Germany). For TEM, ultrathin sections were treated with 3% lead citrate-3H_2O with a Leica EM AC20 contrasting device (ultrastain kit II) and examined at a Zeiss EM109 TEM, equipped with a sharp eye digital camera.

Statistical analysis

The statistical analysis was carried out with GraphPad Prism (version 9.4.1) An analysis of variance (ANOVA) was performed to compare the medians of the different groups. An unpaired t-test was applied to examine the means of two independent groups. To visualize the different intensities of FTH and FTL in NF-Brain and Ctr, intensity values were z-normalized, clustered, and plotted using the R package ComplexHeatmap. [4, 5]

Supplemental Data 5 - Additional References

1. Bankhead P, Loughrey MB, Fernández JA, Dombrowski Y, McArt DG, Dunne PD, McQuaid S, Gray RT, Murray LJ, Coleman HG, James JA, Salto-Tellez M, Hamilton PW (2017) QuPath: Open source software for digital pathology image analysis. Sci Rep 7:16878. doi: 10.1038/s41598-017-17204-5

2. Graham L, Orenstein JM (2007) Processing tissue and cells for transmission electron microscopy in diagnostic pathology and research. Nat Protoc 2:2439–2450. doi: 10.1038/nprot.2007.304

3. Gregory A, Hayflick S (1993) Neurodegeneration with Brain Iron Accumulation Disorders Overview. In: Adam MP, Everman DB, Mirzaa GM, Pagon RA, Wallace SE, Bean LJ, Gripp KW, Amemiya A (eds) GeneReviews®. University of Washington, Seattle, Seattle (WA)

4. Gu Z (2022) Complex heatmap visualization. iMeta 1:e43. doi: 10.1002/imt2.43

5. Gu Z, Eils R, Schlesner M (2016) Complex heatmaps reveal patterns and correlations in multidimensional genomic data. Bioinformatics 32:2847–2849. doi: 10.1093/bioinformatics/btw313

6. Harrison PM, Arosio P (1996) The ferritins: molecular properties, iron storage function and cellular regulation. Biochimica et Biophysica Acta 1275:161–203. doi: 10.1016/0005-2728(96)00022-9

7. Jensen EC (2013) Quantitative analysis of histological staining and fluorescence using ImageJ. Anat Rec (Hoboken) 296:378–381. doi: 10.1002/ar.22641

8. Jumper J, Evans R, Pritzel A, Green T, Figurnov M, Ronneberger O, Tunyasuvunakool K, Bates R, Žídek A, Potapenko A, Bridgland A, Meyer C, Kohl SAA, Ballard AJ, Cowie A, Romera-Paredes B, Nikolov S, Jain R, Adler J, Back T, Petersen S, Reiman D, Clancy E, Zielinski M, Steinegger M, Pacholska M, Berghammer T, Bodenstein S, Silver D, Vinyals O, Senior AW, Kavukcuoglu K, Kohli P, Hassabis D (2021) Highly accurate protein structure prediction with AlphaFold. Nature 596:583–589. doi: 10.1038/s41586-021-03819-2

9. Li H, Durbin R (2009) Fast and accurate short read alignment with Burrows–Wheeler transform. Bioinformatics 25:1754–1760. doi: 10.1093/bioinformatics/btp324

10. McKenna A, Hanna M, Banks E, Sivachenko A, Cibulskis K, Kernytsky A, Garimella K, Altshuler D, Gabriel S, Daly M, DePristo MA (2010) The Genome Analysis Toolkit: A MapReduce framework for analyzing next-generation DNA sequencing data. Genome Res 20:1297–1303. doi: 10.1101/gr.107524.110

11. Paila U, Chapman BA, Kirchner R, Quinlan AR (2013) GEMINI: Integrative Exploration of Genetic Variation and Genome Annotations. PLOS Computational Biology 9:e1003153. doi: 10.1371/journal.pcbi.1003153

12. Varadi M, Anyango S, Deshpande M, Nair S, Natassia C, Yordanova G, Yuan D, Stroe O, Wood G, Laydon A, Žídek A, Green T, Tunyasuvunakool K, Petersen S, Jumper J, Clancy E, Green R, Vora A, Lutfi M, Figurnov M, Cowie A, Hobbs N, Kohli P, Kleywegt G, Birney E, Hassabis D, Velankar S (2022) AlphaFold Protein Structure Database: massively expanding the structural coverage of protein-sequence space with high-accuracy models. Nucleic Acids Research 50:D439–D444. doi: 10.1093/nar/gkab1061

13. NDP.view2 Viewing software U12388-01 | Hamamatsu Photonics. https://www.hamamatsu.com/eu/en/product/life-science-and-medical-systems/digital-slide-scanner/U12388-01.html. Accessed 26 Sep 2022

14. ZEISS Mikroskopsoftware ZEN - Analysieren Sie Bilddaten oder Mikroskopie-Bilder und steuern Sie Mikroskopkomponenten. https://www.zeiss.de/mikroskopie/produkte/mikroskopsoftware/zen.html. Accessed 26 Sep 2022
